# Low cost centrifugal melt spinning for distributed manufacturing of non-woven media

**DOI:** 10.1371/journal.pone.0264933

**Published:** 2022-04-19

**Authors:** Anton Molina, Pranav Vyas, Nikita Khlystov, Shailabh Kumar, Anesta Kothari, Dave Deriso, Zhiru Liu, Samhita Banavar, Eliott Flaum, Manu Prakash

**Affiliations:** 1 Department of Materials Science and Engineering, Stanford University, Stanford, California, United States of America; 2 Department of Bioengineering, Stanford University, Stanford, California, United States of America; 3 Department of Chemical Engineering, Stanford University, Stanford, California, United States of America; 4 Department of Electrical Engineering, Stanford University, Stanford, California, United States of America; 5 Department of Applied Physics, Stanford University, Stanford, California, United States of America; 6 Program in Biophysics, Stanford University, Stanford, California, United States of America; Chulalongkorn University, THAILAND

## Abstract

Centralized manufacturing and global supply chains have emerged as an efficient strategy for large-scale production of goods throughout the 20th century. However, while this system of production is highly efficient, it is not resilient. The COVID-19 pandemic has seen numerous supply chains fail to adapt to sudden changes in supply and demand, including those for goods critical to the pandemic response such as personal protective equipment. Here, we consider the production of the non-woven polypropylene filtration media used in face filtering respirators (FFRs). The FFR supply chain’s reliance on non-woven media sourced from large, centralized manufacturing facilities led to a supply chain failure. In this study, we present an alternative manufacturing strategy that allows us to move towards a more distributed manufacturing practice that is both scalable and robust. Specifically, we demonstrate that a fiber production technique known as centrifugal melt spinning can be implemented with modified, commercially-available cotton candy machines to produce nano- and microscale non-woven fibers. We evaluate several post processing strategies to transform the produced material into viable filtration media and then characterize these materials by measuring filtration efficiency and breathability, comparing them against equivalent materials used in commercially-available FFRs. Additionally, we demonstrate that waste plastic can be processed with this technique, enabling the development of distributed recycling strategies to address the growing plastic waste crisis. Since this method can be employed at small scales, it allows for the development of an adaptable and rapidly deployable distributed manufacturing network for non-woven materials that is financially accessible to more people than is currently possible.

## Introduction

Non-woven materials represent a class of engineered fabrics that are ubiquitous in modern life with applications including apparel, construction, medicine, and filtration [1–3]. In specific, non-woven filtration media have recently received widespread attention for their use in air filtration devices which provide protection against the inhalation of particulate matter [4] associated with increasing air pollution due to industrialization and urbanization [5–7], increasing wildfires associated with climate change [8–11], and most recently to deter the spread of COVID-19 [12–14]. In the context of the COVID-19 pandemic, failures in global supply chains for non-woven materials have inspired researchers and left governments desperate to find solutions to meet global demand surges [15, 16]. Recent research has identified commonly available materials [17–19] that can be used as an improvised face covering while other work has focused on developing effective reuse and decontamination protocols of existing PPE [20–25]. However, little work has been done to address the production bottle-neck of the non-woven filtration material at the center of these devices [13, 26]. Meanwhile, the shortage has been exploited by bad actors who have introduced counterfeit N95 respirators into the marketplace [27] and has led to countries with domestic manufacturing capacity to enforce export controls at the expense of those without such infrastructure, namely low and middle income countries (LMICs) [12, 28–30]. Finally, the problem of managing plastic waste from an estimated daily usage of 6.8 billion masks per day must be addressed in an environmentally friendly manner [31, 32]. These events highlight the importance of rethinking the production and supply chains associated with functional non-woven materials.

**Fig 1:**
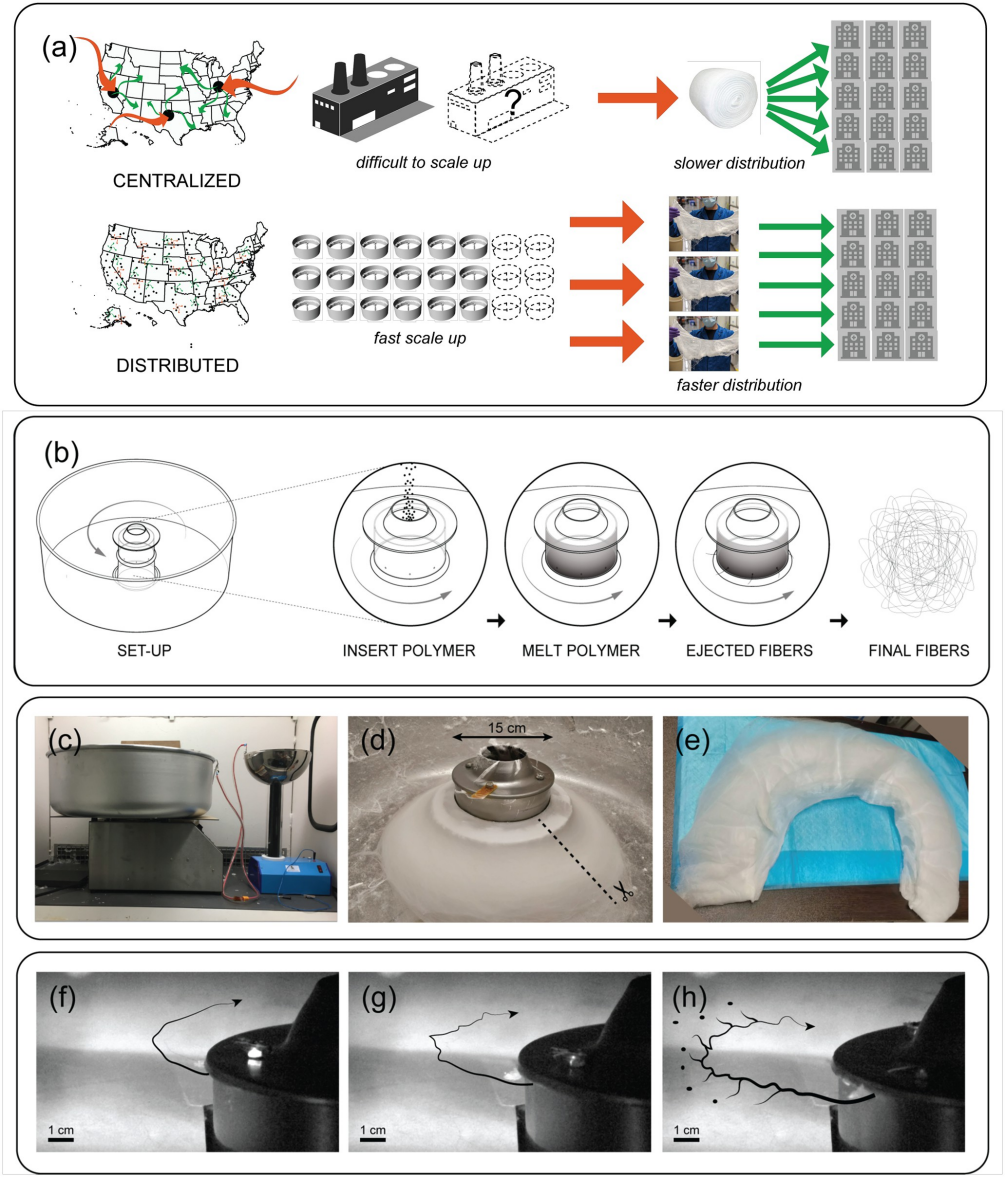
Distributed manufacturing and cotton candy machine for production of non-woven filtration media. A: Distributed manufacturing paradigm allows for flexible, local production of material anywhere in the the world on short notice. B: Schematic illustration showing key steps of RJS process to produce non-woven fiber mats. C: Implementation of RJS using a retrofitted, commercially-available cotton candy machine. D-E: the process deposits fibers in a mat that can be collected and processed into filtration media. F: High speed camera footage showing ejection of material from the spinneret and onset of a Rayleigh-Taylor instability G-H: leading to the formation of nano- and microscale fibers from extrusion holes much larger in size(∼ 500 − 1000*μm*).

Distributed manufacturing (DM) is a framework that relies on geographically dispersed manufacturing nodes operated at small scales to produce goods locally and equitably, offering an alternative paradigm to centralized manufacturing Fig 1A [33–35]. Small- to medium-scale manufacturing nodes are inherently more flexible and resilient than large-scale, centralized production. For example, redundancy in a manufacturing network minimizes the risk of a single point of failure to supply chains. Additionally, manufacturing at this scale requires less capital and time investment, increasing accessibility in LMIC environments and reducing the financial burden of increasing capacity due to surge demand in mature markets [29]. The DM approach has been validated in the context of additive manufacturing where the prevalence of 3D printing and digital design tools has enabled rapid and flexible manufacturing capacity to respond to the present crisis at local scales [36–38]. A DM approach to mask manufacturing was proposed during the initial pandemic response [39]; however, the proposal did not address access to the non-woven filtration material—the main manufacturing bottle-neck. Furthermore, DM provides unique economic opportunities that would be challenging to implement in a centralized model [33, 40]. For example, the development of complementary recycling tools has allowed both new approaches for closed-cycle manufacturing [41] and users to experiment with novel materials from local sources [35, 42, 43]. The development of distributed recycling coupled to DM is a promising route towards increasing efficiency in collection and recycling plastic waste [44–46]. From a sustainability perspective, there is the added benefit of reducing the environmental impact associated with transportation in global supply chains and waste streams [47]. There is a clear need for an analogous technology for the distributed fabrication of non-woven materials that can be used in a variety of applications, including air filtration.

The polypropylene (PP) micro- and nanoscale fibers used in non-woven filtration media are a challenging technical target for distributed manufacturing. The rheological properties of PP allow it to be transformed into thin fibers with dimensions necessary to achieve direct inertial filtration. The dielectric properties of the material on the other hand allow manufacturers to embed electrical charge that aid with filtration in the diffusive range, thus allowing filtration of much smaller sized particles than is possible through inertial filtration alone [1]. The existing supply chain for this material relies on large-scale centralized manufacturing, using a process known as melt-blowing. This process operates by passing hot, pressurized air around a heated extrusion die to simultaneously melt and extrude molten polymer into non-woven fibers. The extensive infrastructure needed for die fabrication and high energy costs required to supply compressed, hot air makes this technique efficient but inflexible to surge demand and economically inaccessible. While a typical melt blowing facility can produce filtration media for more than 1 million masks per day, establishing additional manufacturing capacity requires significant investments of both time (∼ months) and capital (∼ million USDs) [48]. Alternative methods for producing nano- and microscale non-woven fibers that could be operated at small and medium scales include electrospinning and centrifugal melt spinning (CMS) [49, 50]. While electrospinning has benefited from an enormous amount of research, it is still limited by low throughput and an inability to work with low dielectric materials like PP [49]. In contrast, CMS offers an order of magnitude improvement to throughput and is agnostic to the electrical properties of the material, as it eliminates the usage of electrical potential to draw fibers from a polymer pool. In contrast to melt blowing, CMS gains efficiency by decoupling melting and extrusion by using a controllable heating element to melt and centrifugal forces generated by a rotating spinneret to extrude the polymer Fig 1B. Much of the academic work related to the CMS method has used either the FibeRio device [50] or custom-built devices. Several studies have demonstrated fabrication of nano- and microscale PP fibers using CMS [51–53] and even a capacity to produce fibers from commonly available mixed recycled plastics [52]. Indeed, CMS has been used as an enabling technology for distributed recycling efforts [54]; however, the possibility of creating higher value-add, functional materials has not been rigorously discussed in the academic literature.

Here, we demonstrate that CMS can be implemented with simple hardware and used to obtain functional non-woven materials in a way that is commensurate with the requirements of DM. Specifically, we use a modified commercially-available cotton candy machine (CCM) to show that CMS is both a fast and affordable means to produce non-woven materials. In our investigation, we consider the use of different resins on the morphology and performance of the resulting non-woven materials. We explore strategies required for processing the produced material into a functional fabric and for performing quality control in a distributed manufacturing context. The performance of our functional fabrics is compared with commercially-available N95 filters and evaluated according to filtration efficiency (FE) and pressure drop (PD). The purpose of this work is to contribute to the ongoing discussion concerning the limits and opportunities of small- and medium-scale manufacturing for the production of medical equipment and—more generally—technologically advanced materials such as non-wovens.

## Results

### Centrifugal melt spinning

Commercial CCMs consist of an electrically heated spinneret with a material reservoir that is spun using a vertically mounted electric motor Fig 1C [55]. The spinneret is rotated while being simultaneously heated to generate the centrifugal forces necessary for the extrusion of the molten material contained within. The molten polymer extrudes through tiny orifices in the spinneret in a radially outward direction in the spinneret’s frame of reference. The molten polymer mass undergoes an extensional flow and thins out into smaller diameters [56]. Simultaneous cooling due to ambient temperature gradients and surrounding air flows causes solidification of the stretched melt flow, which ultimately defines the diameter of the resulting fiber. These fibers then accumulate as a sheet on the walls of the cylindrical enclosure around the spinneret.

With all the necessary principles for CMS present in the CCM, we decided to modify such a device by replacing the wire screen on the spinneret with a solid aluminum ring having several orifices (8–24) with uniform diameters ranging from 0.016”—0.038” (0.4064–0.9652 mm) (S1 Fig). These orifices allow for well-controlled extrusion of molten polymer and allow us to obtain a fiber diameter range required for filtration media applications (0.1–10 *μ*m). To prevent self spooling of extruded fibers by the spinneret shaft, we introduced a simple cardboard cover shield, which allowed formation of continuous flat fiber sheets on a conical surface around the spinneret Fig 1D. Fibers accumulate as an annular sheet with the inner circumference adhered to the spinneret and the outer circumference adhered to the collection cylinder. The material used for further testing is obtained by cutting open the sheet radially and flattening it out on a surface to remove regions of the sheet which display defects associated with static collection during batch assembly. We observe two types of processing defects. First, material near the outer circumference has a lower density due to a constant mass flux being deposited over an area which scales with *radius*^2^. Second, material near the inner circumference becomes fused due to its close proximity to the heated spinneret. The material collected between these two defect regions is shown in Fig 1E and was extruded in ∼2 *min*. and weighs ∼ 24–26 *g*, containing enough material for ∼ 12 − 13 masks.

Through high speed imaging of the machine in operation, we also observed the breakup of pre-solidified jets into smaller droplets due to the presence of a Rayleigh-Plateau instability, often resulting in the formation of microspheres. We consider the presence of microspheres to be a source of contamination for filtration media since they can be dislodged by air currents. This can be controlled by using higher viscosity or higher surface tension materials, or utilising electric fields for drawing fibers (Fig 1F–1H), as has been reported previously [51, 57, 58]. Another source of microsphere formation is the breaking up of a polymer flow stream at the orifice tip itself. Having a replaceable aluminium ring design allowed us to easily debug and tune the geometry of the fiber being produced by simply swapping the ring with a different orifice size. In general, we observe that larger orifices lead to larger diameters; however, we finally settled on an orifice size of 0.024 inches for the data presented in this study.

One of the limitations of using a commercially-available CCM, is the lack of precise temperature control and access to only a single RPM value. As a result our study considers fibers produced at *T* = 160 − 200°*C* and 3,500 RPM. This range in measured temperatures is a result of a changing amount of material in the reservoir. As material is depleted, the constant-voltage heat source continues to provide the same energy input leading to higher observed temperatures.

Application of an electric field during the fiber extrusion process has been used to minimize the production of microspheres [57], reduce fiber diameter, and to impart an electric charge to the material—which is a common strategy for increasing the FE of the non-woven material [59–61]. The presence of an electric field during fiber extension has also been shown to produce charges embedded within the fiber volume that are more stable against environmental conditions such as humidity and temperature compared to surface charges [59, 62]. To implement similar strategies, we connected a -5kV potential source with the negative terminal placed on the collection drum. Since the spinneret was electrically grounded, a field was established with the spinneret at ground potential and the collection drum at -5kV negative potential, allowing for polarization of the extruded molten polymer.

We characterized the charge of the material with a handheld, electrostatic surface DC voltmeter. The readings of the voltmeter only provide a crude description of the charge distribution on the surface of and inside the material [63]. Measurements performed right after the material is produced lead to the most consistent readings ranging from -1 to -10 kV. The surface potential is relatively uniform (varying about 30%) along the surface and is similar on both sides of the collected fiber sheet. We also observed that surface contact with other dielectric materials used for handling and storing the fibers lead to high variance in the charge measurement of the sheet. Such large variance is similar to previous work in the literature using similar characterization methods [64]. This suggests that most of the collected charge was either able to conduct or get exchanged through triboelectric charge transfer. The low charge retention could be attributed to the conduction properties of the material, predominantly surface accumulation of charges instead of the bulk or the energy level of the localized bulk charge trapping sites [65, 66]. We tested this hypothesis by utilising a custom made corona discharge device using a van de Graaff generator and friction induced triboelectric charge transfer from commonly available polystyrene packaging material. Such treatments resulted in temporary enhancement of surface charges which have been recently utilized for rejuvenation of filtration properties post decontamination of N95 masks [64, 67–69], but the material maintained aggressive charge exchange properties with the surfaces in contact.

### Fiber morphology and processing

**Fig 2:**
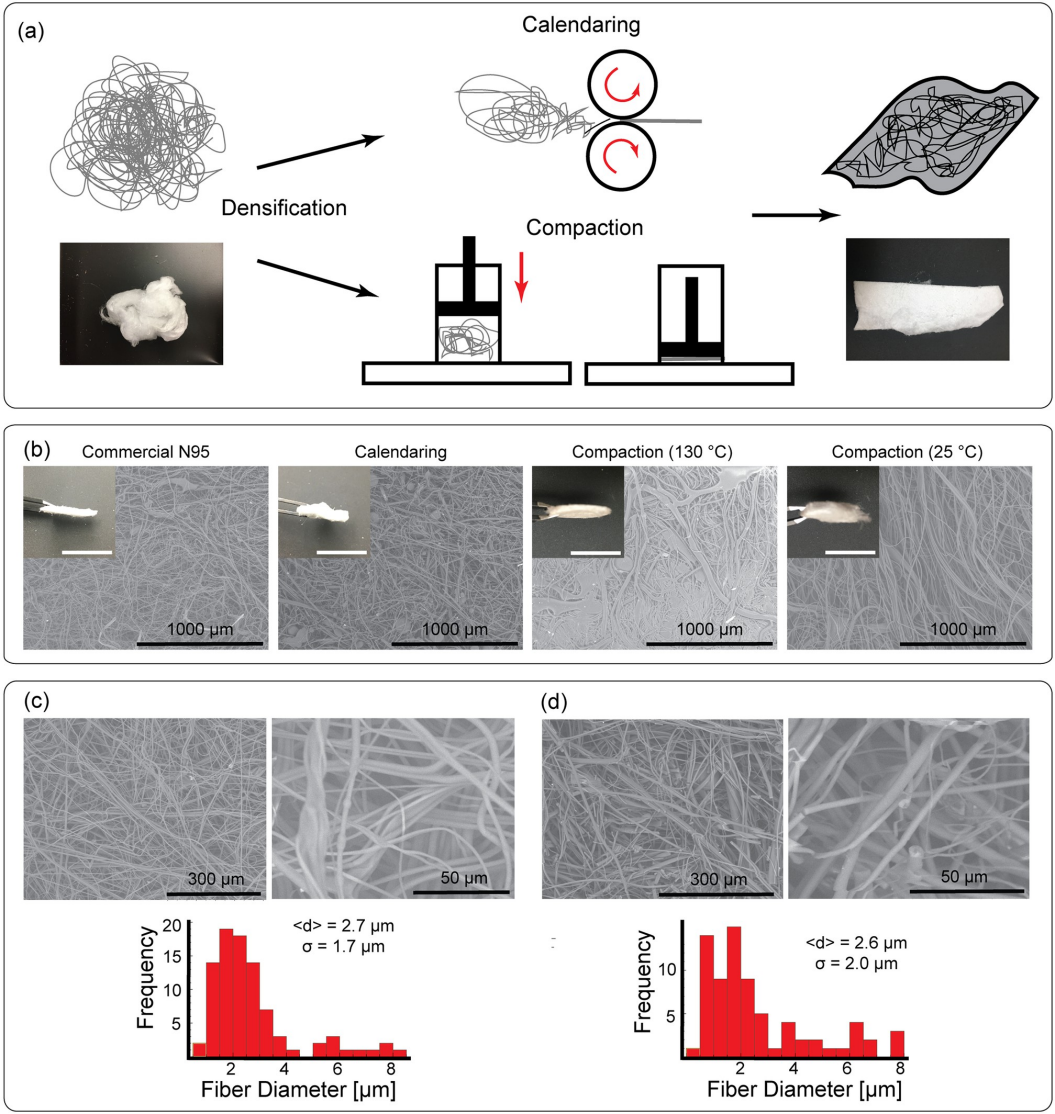
Fiber processing and characterization. A: Produced fibers must be processed into dense mats before they can be used as a filtration media. We evaluate two methods: i) calendaring and ii) compaction. Photographs show as-produced material (left) and material after compaction (right). B: SEM characterization of large-scale features of non-woven filtration media produced using Pinnacle 1112 PP homopolymer (MFI = 12 g/10 min). Insets show a macroscopic section of material obtained after each densification process compared with material obtained from a commercial N95 mask; scale bar represents to 1 cm. C: SEM characterization (top) enables comparison of fiber morphology between commercial N95 and fibers produced from using a modified CCM (image obtained from calendared sample shown in part B). Histograms (bottom) of fiber diameters show that both samples share a similar long-tailed distribution of fiber diameters. The black curve is a continuous probability distribution derived from the experimental data. Insets show the same distribution plotted on a logarithmic axis. Fiber diameters were measured from the sample at several different locations using 150 fiber counts.

As compared to the fiber sheets obtained through the commercial melt-blowing process, those obtained through our method have lower density of fibers and hence require additional post processing before they can be evaluated as candidates for air filtration media, an approach that is distinct from one of the recent studies in this space [26]. Here, we consider two approaches for densification: 1) calendaring and 2) compaction. We compare the fiber morphology of the resulting materials against commercially-available N95 FFR filter media (Fig 2A and 2B) through scanning electron microscopy (SEM). Our calendaring process was carried out using cold lamination rollers. The material is supported between two layers of spun-bound PP to prevent adhesion of the material to the rollers. The support layer is then removed for subsequent testing. The resulting material shows an increase in density but still lower than that of the reference material. Meanwhile compaction was carried out with and without the application of heat (130°*C*). We find that application of heat is important to increase the density of the material and produces a densification similar to that of the reference material. Compaction without heat produces the least dense of the samples considered here. Both of these techniques allow for the construction of multi-ply filters which makes them more mechanically robust. Additionally, multi-layer constructions of filtration media are important since a failure in a single layer will not lead to a failure of the entire filtration device, since a defect at a position in one layer can be compensated by continuous material deposition in the other layers.

Comparison of the material microstructure (Fig 2B) provides further insights. We find that the sample subjected to calendaring is most similar at the microscale to the N95 reference material. Heat compaction produces a fusion of the fiber network which results in extremely large pressure drops (data not presented). This can be understood by noting a significant reduction in pore size compared with the other samples in our study. Reduction in the applied shear forces during calendaring also contributes to preserving randomness in the fiber network, which has been realized by industrial processes for ensuring high FEs through complex carding processes [70].

We finally examine the morphology of individual fibers for calendared samples (Fig 2C). We see that the N95 reference material has an average fiber diameter of 3.7 ± 2.6*μm*. However, the distribution of fiber diameters shows that while most of the fibers are on the single micron scale, the distribution extends to include much larger fibers. This wide distribution allows for larger fibers to act as a support for the smaller fibers which are typically more fragile. The fibers produced by our method using PP resin with *MFI* = 12*g*/10*min* have slightly larger average fiber diameters (3.4 ± 3.0*μm*) and a similar long-tailed distribution that is skewed more heavily towards large diameter fibers. These morphological similarities with the reference material at all length scales make these materials promising candidates for use as air filtration media. Additionally, we processed PP resins with increasing *MFIs* which are typically used in traditional melt-blowing manufacturing (S3 Fig). We find that resins with *MFI* = 50, 500, 1550*g*/10*min* give fibers with diameters 5.2 ± 3.8*μm*, 4.1 ± 3.9*μm*, and 3.8 ± 5.2*μm*, respectively. In general, increasing MFI results in fiber diameter distributions that have a higher fraction of small diameter fibers (∼ 1*μm*).

However, we note the presence of artifacts associated with batch processing. The continuous sheet of fibers collected after a run shows variations in the fiber diameters with the size of the fibers decreasing with newer fibers deposited on upper layers. One possible explanation for this is the variable amount of heat absorbed by the material depending on the time it spends in the spinneret. The material to extrude last suffers from higher polymer decomposition and has altered mechanical properties. Moreover, smaller amounts of material in the spinneret also prevents the orifices from being completely filled during the extrusion, which reduces the effective orifice diameter participating in the extrusion, thus altering the nominal diameter and mechanical properties of the fibers expected during a run.

### Material performance

**Fig 3:**
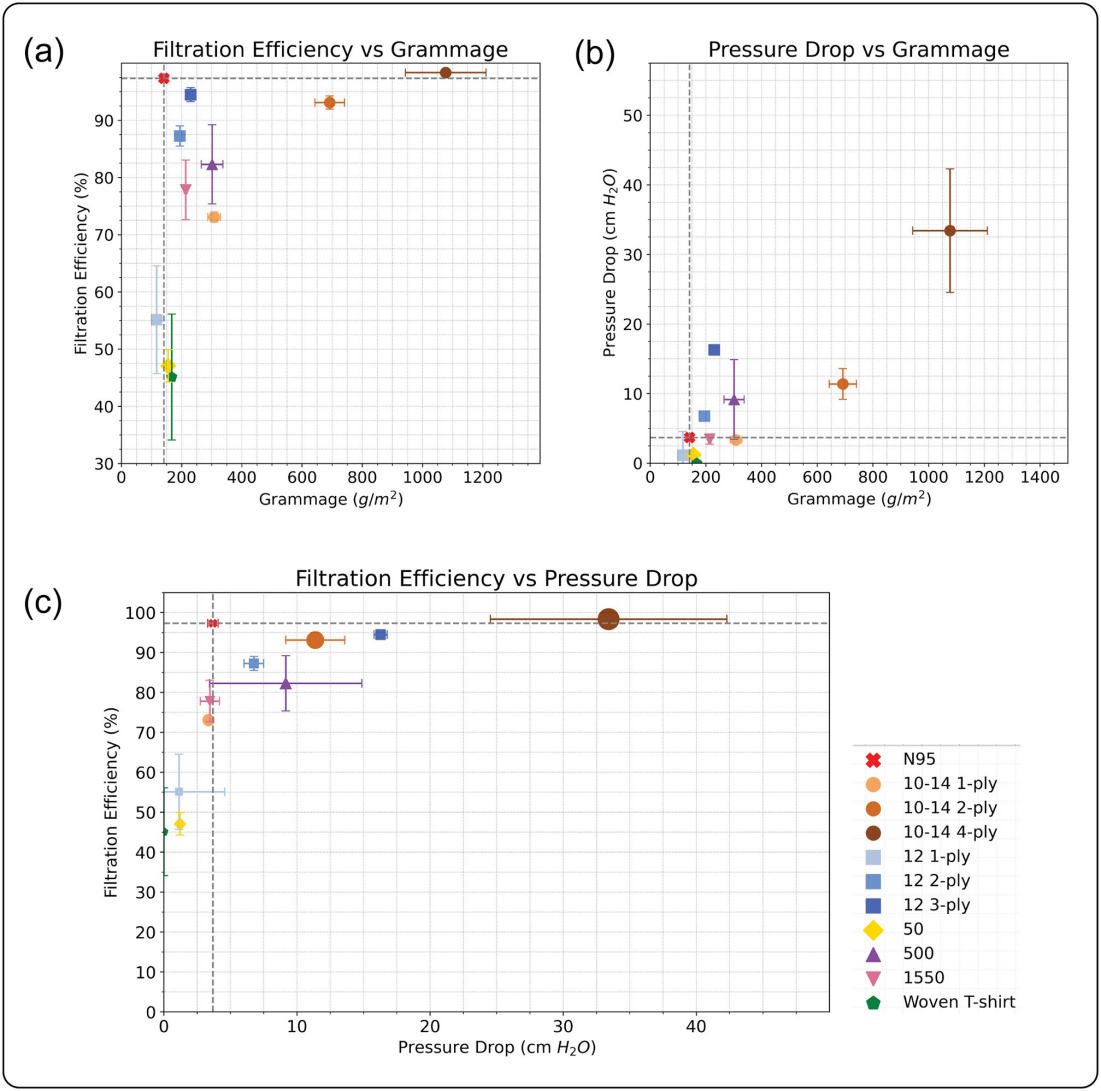
Performance of non-woven filtration media. A: Filtration efficiency and B: pressure drop for several different materials produced via CMS plotted against the grammage of the sample. The numbers in the legend indicate the melt flow indices of the polymers. C: Phase plot of filtration efficiency vs pressure drop with marker size representing grammage of the sample. The markers represent mean reading from *N* ≥ 3 samples with a triplicate experiment for each sample. The error bars represent standard error of the mean on each side for both vertical and horizontal axes. The dashed lines represent the corresponding measurements for the filter material extracted from N95 FFRs. All the samples were prepared using 30*g* of polymer material except for those with explicitly mentioned values of 12*g*.

We evaluated the FEs and PDs of the processed materials using a setup similar to that used in previous studies on filtration materials (S2 Fig) [71]. FE measurements were made using incense smoke as a source of particles with a range from 0.01 to 5.0 *μ*m with a flow rate of 2.8 *L*/*min*. This rate is set by the particle counter (S2A Fig). We note that this flow rate is substantially lower than what is specified by conventional testing standards [17, 20]. PD represents the air resistance across the filtration media with lower values indicating higher breathability. All measurements were made with a flow rate of 5 *L*/*min*. Samples are excised from calendared material with diameter 17.25 mm (S2B Fig). Under these test conditions, we show that several of our produced materials have FEs comparable to the N95 reference material of the same dimensions subject to the same experimental conditions (Fig 3A). In specific, the N95 sample had a measured FE of 97.34 ± 0.57% compared with 94.48 ± 1.23% of our best material (*MFI* = 12*g*/10 *min*, 3ply). While several of our samples performed quite well, there are important trade-offs in material performance that are dependent on material processing. For example, our best material (*MFI* = 12*g*/10*min*, 3ply) has a pressure drop of 16.28 ± 3.43 *cm* *H*_2_*O* which just underperforms that of the N95 reference 3.69 ± 0.39 *cm* *H*_2_*O*. However, by reducing the number of plys in the construction, we can reduce the pressure drop to 6.76 ± 0.74 *mm* *H*_2_*O*/*cm*^2^ with only a slight sacrifice in filtration efficiency 87.26±1.77%.

To better understand the design space associated with material processing, we consider the effects of density on both FE and PD. We find that density does not have a significant impact on FE (Fig 3B). However, we do observe a significant reduction in FE for single ply samples with decreasing density. We can understand this observation by recognising that any defects in the non-woven material will lead to a failure of the filter. This failure mode can easily be remedied by adding a second ply which allows defects in one layer to be compensated by functional regions of the other layer. We also considered the effect of the relative orientation of sheets for two-ply filters. We find that there are no significant effects, confirming the presence of sufficiently random fiber orientations observed in the microstructure of single layers. We also observe a significant dependence of PD on density. Increasing density results in reduced breathability. Our data suggests that two-ply materials find a good balance between FE and breathability. More generally, we have shown that a modified cotton candy machine can be used to produce functional fabrics which might be useful in air filtration applications. While the performance metrics are less than those of the N95 reference material, they are comparable with those of community-based mask manufacturing efforts [72].

### Distributed manufacturing and recycling

**Fig 4:**
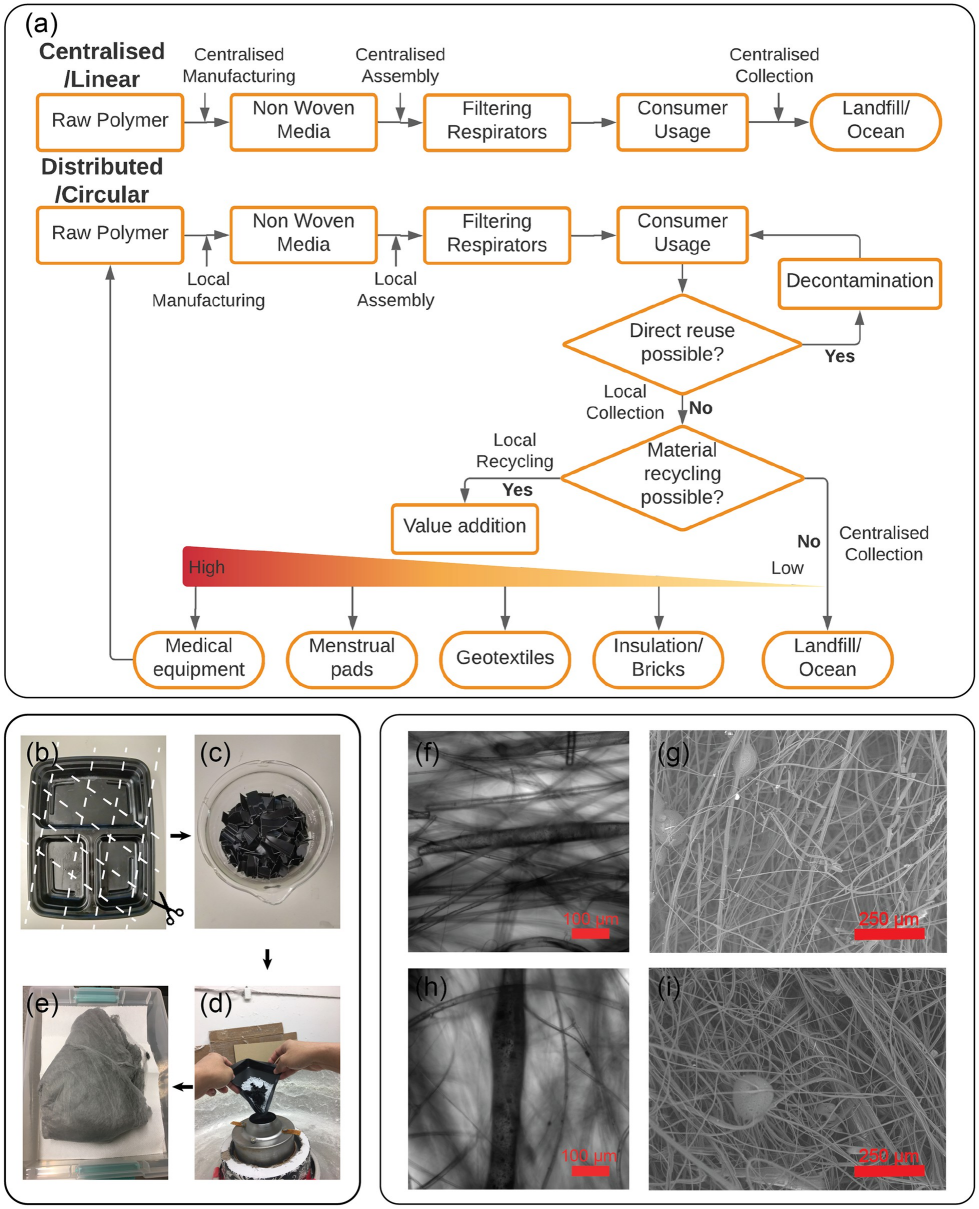
Incorporation of locally sourced recycled material. A: A distributed manufacturing framework enables incorporation of cycles within material life from raw polymer to dumping sites at landfills or oceans. Access to machines like the one presented in the study allows addition of value during recycling process, improving the chances of the material to be reused for multiple applications during its lifetime. B-E: Locally sourced waste polypropylene was cut into small pieces and combined with virgin 1112 PP resin at 1:5 ratio to produce fiber sheet. F-H: Light micrographs of thick fiber stems at 30x magnification. Phase separated droplets of waste material are visible inside as dark inclusion, which increase in number as we go from 20% (F) to 80% (H) proportion of recycled polymer. G-I: SEM characterization of the recycled-PP/PP hybrid showing unnoticeable variation in fiber morphology as we go from 20% (G) to 80% (I) proportion of recycled polymer.

Having shown that functional fabrics can be produced using relatively simple hardware, we now explore what this implies for distributed manufacturing and recycling of plastics, specifically non-woven materials (Fig 4A). A key problem associated with the manufacture of plastic-derived materials is their collection and disposal. Currently, most waste streams involve international supply chains, adding to their carbon footprint and creating friction due to evolving international trade agreements [47, 73]. However, in many other regions, such waste streams are either non-existent or poorly managed [32]. As a result, waste often ends up in the environment. This problem has been exacerbated by the ongoing COVID-19 pandemic which has seen not only an increase in plastic waste from single-use medical equipment [32] but also an increase in single-use packaging material [74]. By distributing tools for plastic processing, an economic incentive is created to more efficiently collect plastic since this material can be re-purposed or up-cycled [73]. Currently, most tools designed to be operated at local scales focus on either additive manufacturing (e.g. 3D printing) [36] or extrusion based technologies [46]. To the best of our knowledge, Polyfloss is the single example of an effort using CMS technology for recycling, operating as a grassroots organization to transform waste plastic into building insulation [54]. The potential for more sophisticated non-woven fabrics has not been explored.

Here, we show that plastics from consumer waste streams can be incorporated into the non-woven production process. In general, the processing of polymer blends or even differing grades of the same polymer can pose challenges, particularly mixing of polymers with different thermal and rheological properties. Furthermore, multi-generation processing (e.g. processing the same plastic multiple times) can lead to changes in material properties [41]. As a simple demonstration that the process presented in this report can empower people to experiment with sustainable, closed-cycle manufacturing, we show that a fraction of the virgin PP resin can be replaced with PP obtained from consumer waste (Fig 4B–4E). At low fractions (1:5), we observe inclusion of phase separated droplets of recycled material within the solidified fibers from virgin polymer [75]. SEM analysis shows that the material also contains more microspheres than found in material prepared from unmixed, virgin polypropylene (Fig 4F–4I). As the fraction of recycled polypropylene is increased, the presence of microspheres increases but the fibers are similar in morphology to those made from lower fraction blends (Fig 4G–4I), indicating saturation towards mixing between the polymers.

The scalability of CMS is a key factor in assessing its viability as the technological basis for distributed manufacturing. Rogalski *et al*. report that a lab-scale CMS device can produce up 60 g/h of material per orifice (roughly 100 times greater than typical rates for electrospinning) [49]. Our experiments are in good agreement with these rate estimates across several different implementations. Given that a typical N95 FFR contains ∼ 2g of filtration material, we estimate that a rotating chuck with a single orifice can produce material sufficient for 500–1000 N95 FFR in a 16 hour day if operated continuously. We set an upper estimate of the motor, heating element, and metal chuck to cost $1000 USD and can be set up in at most several days. We note that increasing the number of orifices per chuck and operating multiple chucks in parallel can offer significant improvements over this lower bound on throughput. The produced material must be assembled into a functional FFR. When coupled with a local mask production effort [72, 76], a few devices operated in parallel can provide a flexible and significant surge capacity for a local community.

The advantages of this approach become apparent when compared with the time and capital cost of establishing a conventional melt-blowing facility. Such a facility costs 0.1 − 1 million USD and requires several months to bring production capacity of ∼1 million masks/day online [48, 77]. A similar capital investment of 1 million USD would enable the establishment of a distributed network of 1000 CMS setups, which, at a rate of 1000 masks per day per setup would successfully match the output of a centralised melt blowing facility. It is important to note that this comparison reveals that the ratio of capital investment to mask output is equivalent for the two manufacturing models. What is gained is the ability for rapid deployment, supply chain resiliency, and increased accessibility. Since the required hardware can be readily sourced or manufactured on-site using digital fabrication tools [78], surge capacity can be quickly brought online in areas where it is needed the most during times of crisis without being a financial burden for existing manufacturers. Furthermore, operating at a smaller scale lowers the capital burden required for operating this manufacturing capacity. This can enable domestic manufacturing capacity in LMICs where export restrictions and market competition has made access to reliable filtration media prohibitive and provide flexible surge capacity in mature markets in times of crisis.

We thus envision the ideal life cycle of a mask during an emergency response to be as follows (Fig 4A). Polypropylene is sourced locally [79] by micro-factory operators who convert raw polypropylene pellets into non-woven material using a small scale production tool like the one described here. Typically, a face filtering respirator consists of a filtration layer separated by two support layers. The filtration layer is produced from high MFI PP (> 1000 MFI); however, appropriate grades of polylactic acid have recently been shown to be effective—pointing the way toward a potentially broader set of candidate materials [80]. Meanwhile the support layers are produced from low MFI PP (<100 MFI) characterized by much larger mean fiber diameters. The ability to process multiple grades of polypropylene spanning several orders of magnitude point the way towards producing both support and filtration layers using the same device. However, we anticipate that this will require careful tuning of operating parameters, specifically temperature and rotation speed. These produced materials can then be processed into a device using community volunteers [72, 76]. Depending on how the micro-factory is financed, the produced masks can be freely distributed to the community or sold to recoup capital expenditures. After the mask has served its useful life, the masks can be collected at the same scale at which they were produced. To create an incentive for collection, a deposit can be associated with mask return. Numerous reports have emerged concerning proper handling of used masks that would render them safe to handle [23–25, 81]. For example, heat treatment at 50 C for 20 mins [23] or treatment with 1% NaOCl [24] The collected masks could then be recycled and re-purposed. Here, there are numerous possibilities. In one scenario, masks could be incorporated with virgin resin to make new masks with lower raw material input. Alternatively, the filtration material could be used in a more general application, e.g. building air ventilators. There are also value-added applications which have very low material standards, examples include building insulation [54] or concrete [32]. The key idea is that the ability to locally repurpose the material will create an incentive to reduce waste leakage into the environment as it eliminates energy and resource expenditure for identification and segregation which would be required at centralized dumping sites [73]. Of course, this framework is quite general and not just limited to face masks but can be applied to a wide range of plastics beyond the crisis response by extending what can be done locally beyond collected plastics using additive manufacturing with 3D printers.

## Discussion

In this study, we have shown that with relatively minimal hardware requirements, non-woven materials with an air filtration performance comparable to commercially-available products can be produced. We achieved this by modifying an existing CCM design that works on the principle of CMS. Although CMS has been a known approach to process molten polymers into fibers, this study demonstrates the capability to produce a functional PP filtration media using CMS for the first time. However, consistent production of high quality material through such a setup will require optimization and further process design. For example, in some instances we observe decomposition of PP resin and the production of brittle fibers. Both of these observations highlight the importance of thermal management during processing. Brittle fibers can result from either an increased crystalline content within the material or from the introduction of defects the production of which are highly temperature dependent processes. This issue can be addressed by incorporating feedback into temperature control (e.g. PID control) or by operating the device in an enclosed chamber. The former will also likely address the issue of polymer decomposition. Another approach to address this issue is to operate the device in a continuous manner, thereby ensuring that the thermal load is constant and minimizing temperature fluctuations. This also has the advantage of increasing throughput and ease of operation. An alternative approach might be to use lower viscosity polymer resins that will be ejected into fibers at higher rates at a given temperature. Previous reports suggest that microsphere contamination can also be addressed through more stringent temperature control or by increasing polymer flow rates [51]. The rotation speed of the spinneret is another control parameter that might provide access to wider range of fiber morphologies and increased throughput [3]. Meanwhile, polymer additives can be used to address some of the other issues encountered in this work. For example, ionic surfactants can be incorporated to help retain electrostatic charging while interfacial energy reducing agents can be employed to improve mixing between recycled and virgin polymers. More generally, access to low-cost tools will accelerate the rate of innovation in polymer processing. For instance, the proliferation of 3D printing has allowed for a broad range of new composite materials that leverage waste streams or unique, local resources to produce new filament materials, including composite materials.

## Conclusion

In this study, we have demonstrated that CMS can be performed using simple hardware to produce non-woven plastics from both raw plastic feed stock and consumer waste streams. The specific implementation in this paper was realized by repurposing a commercially-available cotton candy machine to produce air-filtration media. After appropriate processing, the produced materials consistently exhibit filtration efficiencies near those of the reference material. Additionally, we show that it is possible to process consumer waste streams into non-woven materials, enabling the possibility that distributed manufacturing and recycling can be realized in the same device. The physical mechanism underlying fiber production can be implemented at significantly reduced capital and time investment compared with existing manufacturing techniques, providing an elastic manufacturing capacity that is critical during times of crisis. Moreover, the flexibility made accessible through a shift in manufacturing strategy has allowed us to probe a broad range of materials and quickly test their properties which would have been impossible with a centralised large scale manufacturing unit.

The next direction for this work is to develop an open-source tool which will enable the manufacturing of a wide range of non-woven media for a variety of application. The existence of such a tool will help to realize a distributed manufacturing network whose nodes can be operated by a far broader set of communities than is currently possible. While we have demonstrated that this approach is technically feasible and can be scaled, there are regulatory and organizational challenges that must be met. Similarly, developing a regulatory framework that can accommodate decentralized manufacturing has been a key challenge for the field of open manufacturing as a whole. While concerns have been raised over the ability of small scale manufacturers to produce high quality goods, we see this a challenge to be overcome rather than a fundamental obstacle. It remains an open challenge to reconcile these tools and approaches with standards set by regulatory bodies, particularly in developed countries [33]. By integrating this device with appropriate quality control testing tools, we aim to create a rapidly deployable factory-in-a-box that can be operated even in resource constrained environments to produce non-woven filtration media on short notice. To this end, we have launched an open-source initiative called Project 1000-by-1000 with the ultimate goal of producing a tool that can operate in a continuous manner [82].

This work is important because it provides an alternative to a brittle supply chain that has left a large portion of the global population under served. The flexibility of the underlying method allows for a variety of materials to be reused, including discarded facecoverings or any waste plastic with the requisite physical properties, allowing application of closed-cycle manufacturing thinking to a new class of materials, namely non-woven plastics. The current set of applications for non-woven materials beyond FFRs is incredibly broad, ranging from industrial ventilation filters [1] to feminine hygiene [2]. The case of feminine hygiene products deserves more discussion since similar issues around manufacture, distribution, and waste management stand to benefit from innovation in technology enabling distributed production of non-woven materials [83]. Indeed, distributed, local manufacturing has been identified as a promising route since it minimizes the effects of high import tariffs associated with foreign produced goods. However, existing decentralized, local manufacturing suffers from low-quality standards, inefficiency, and low through-put. While our work focuses on the production of polypropylene melt-blown fibers, the same technology represents a promising approach for manufacturing materials that might be useful in feminine hygiene products with a higher quality and throughput than is currently possible. More generally, access to a low-cost manufacturing device enables distributed experimentation and adaptation, empowering people to discover new materials to use in traditional non-woven applications or to identify new applications for non-woven materials altogether that can be shared in open access online databases [84]. This project was inspired by a failure of conventional supply chains to provide a critical material needed for FFR manufacturing, but we anticipate that the utility of the presented approach will be valued well after the on-going COVID-19 crisis has subsided.

## Materials and methods

### Polymer materials

Polypropylene of different melt flow rates (MFR) were acquired and used without modification: Sigma Aldrich isotactic polypropylene *M*_*w*_ ∼ 250, 000 (MFI = 10–14 g/10 min), Pinnacle polypropylene 1112 (MFI = 12 g/10 min), Ineos polypropylene 100-CA50 (MFI = 50 g/10 min), ExxonMobil Achieve^™^ Advanced PP6035G1 (MFR = 500 g/10 min), and ExxonMobil Achieve^™^ Advanced PP6936G2 (MFI = 1550 g/10 min). Commercial N95 FFR used as reference material was obtained from a Kimberly-Clark 62126 Particulate Filter Respirator and Surgical Mask (Kimberly-Clark Professional, Roswell, GA).

### Cotton candy machine

We purchased the Spin Magic 5 quick release head cotton candy machine (Paragon Inc., USA) and performed the following modifications to its design to achieve the production of flat fiber sheets. Firstly, we replaced the cylindrical wire mesh on the spinneret with a solid aluminium cylindrical ring of identical dimensions (15cm x 4.5 cm, 1mm thick) (S1A Fig). The aluminium cylindrical ring was also pre-drilled with holes around its circumference in the diameter range 0.016” − 0.038” (0.4064–0.9652 mm) to create pores for melt extrusion. Secondly, we utilised layers of Kapton(polyimide) film tape to create a tight heat-resistant seal between the aluminium ring and the contact surfaces with the top cap and the base of the spinner, to prevent any leakage of the polymer melt (S1A Fig). Finally, we fitted a cylindrical cardboard covering to shield the gap between the rotating spinneret and the motor cap at the bottom, to prevent any spooling of produced fibers from the spinneret on the motor shaft itself (S1B Fig). This allows fibers to deposit in spiral pattern forming a conical sheet manifold. This deposited material is cut open to make a flat sheet (Fig 1D and 1E). In order to create an electric field between the spinneret and the collection surface, we attached a -5 kV potential source (Model PMT2000, Advanced Research Instruments Corp.) with the negative terminal placed on the collection drum.

### Temperature measurement

The commercial cotton candy machine comes with an in-built voltage controller for varying the heat being delivered to the spinneret. The current is supplied to the spiral heating element within the rotating spinneret through two carbon brush based rotating connections. In order to melt polypropylene (melting temperature 160°C) efficiently and without much decomposition, the machine was set to a heat setting of 7–8, which resulted in the temperature ranging between 160°C—200°C depending on the running status and the amount of polymer mass available for melting. The temperature measurements were done using a FLIR One Pro LT smartphone module infrared camera and Etekcity Lasergrip 800 Digital Infrared Laser Temperature Gun.

### Surface voltage measurement

All surface voltage measurements were performed using a commerical SVM2 surface DC voltmeter (Alphalab Inc., USA). The surface charges were then calculated based on the measured voltages. Most ideal measurements can be done for infinitely large sheets, but we approximated the measurement process by using smaple sheets with a size of at least 10 *cm* by 10 *cm*. Samples were kept 2.54 *cm* away from the sensor for the measurements.

### Densification of non-woven filtration media

Densification of the produced fibers was performed either by compaction or calendaring. Compaction was performed both with and without heat. Briefly,the uncompacted material was loaded into a cylindrical pipe and pressed against a heated metal plate (130°C, 30 s, 4.23 kg cm^−2^). Calendaring was carried out without heat by feeding single or multiple sheets of uncompacted material through rollers (VEVOR 39” Hand Crank Pressure Cold Roll laminator) lined with spun-bound PP to prevent exfoliation through material adhesion with the roller surface. Layering of the raw material allowed for the fabrication of multi-ply filters. In this study we tested filters with up to 4-plies. Three independent replicates were prepared for each sample tested, sourcing from the same batch of material produced for each of the different PP resins. Circular samples (17.25 mm diam.) for filtration testing were excised from this compacted material.

### Scanning electron microscopy

A Hitachi S3400N SEM operated at 5 keV was used to obtain the micrographs. The compacted fiber samples were attached to the stage using conductive silver paste. and sputter coated Au/Pd (60:40 ratio). Histograms of fiber diameters were produced by measuring multiple locations from the same sample; 150 fiber diameters were extracted from SEM micrographs using the ImageJ package (version 1.52q).

### High speed imaging

We performed high speed imaging of the centrifugal melt extrusion process using Phanton VEO 640S high-speed camera along with a Tamron 70–300 F/4–5.6 Di LD, Model A17. The field of view was illuminated using a 10000 lumens LED flashlight.

### Filtration efficiency measurements

The filter efficiency testing is done using a custom experimental setup made using a handheld particle counter (Model 3016 IAQ, LightHouse, USA) and a sample holder cartridge made from pipe connectors (universal cuff adaptor, teleflex multi-adaptor) (S2A Fig). Whereas, a typical testing setup uses an all-in-one filter tester, *e.g*. 8130A automated filter tester (TSI Automated, USA), that supports a flow rate up to 110 L min^−1^, our system was run at an airflow rate of 2.83 L min^−1^, which was limited by the flow rate provided by the handheld particle counter.

We tested two approaches for generating particles for filtration measurement. In the first one, we used a burning 100 g incense as particle source (Nag Champa, Satya Sai Baba, India). The incense produces particles of various sizes, including those in the range picked up by the detector (0.30–10 μm), and primarily in the 0.30–0.49 μm range. A similar set up had been used in previous studies of air filters [71]. In the second one, we repeated the measurements with particle counts directly collected from room air (without using an incense source) as suggested by Leite et al. [85] and were able to achieve similar results. Since the second approach was much simpler, we only report results from the second approach.

Each sample was tested in triplicates and at least three unique samples were tested. Circular discs of diameter 17.25 mm were cut from the calendered filtration media sheets and sandwiched between holed acrylic supports and sealed on the sides using Parafilm tape. The effective exposed area of the samples through the acrylic supports was 0.291 *cm*^2^. The cartridge was then securely locked in a pipe connector assembly as shown in S2B Fig. The filtration efficiency is calculated as follows, using the 0.3 *μm* particle count from the sensor readings -

$$\begin{array}{c} Filtration\;Efficiency=\frac{\left(n_{without\;filter}-n_{with\;filter}\right)}{n_{without\;filter}}\times 100 \end{array}$$ (1)

Where *n*_*without filter*_ is the particle count without the filter and *n*_*with filter*_ is the same with the filter.

### Pressure drop measurements

For all the pressure drop measurements presented, we utilized volumetric flow rates that resulted in face velocities comparable to what has been used in the literature. For our analysis, we followed reference flow rate values of 30 *L*/*min* used for a sample diameter of 40 *mm* [86]. For these values, the face velocity comes out to be 23.88 *m*/*min*. In order to match these velocities for our exposed sample area of 0.291 *cm*^2^, we required a flow rate of 0.698 *L*/*min*. Hence, we performed all our experiments with compressed air flow velocities just right below this value, varying in the range 0.56–0.62 *L*/*min*.

The same sample-containing capsule used for filtration testing was also used for pressure drop measurements. The airflow rate was measured using a Mass Flow Meter SFM3300 (Sensirion AG, Switzerland) and the pressure drop was measured using a Honeywell ABP Series pressure sensor (Model ABPDANN005PG2A3, Honeywell International Inc., USA). Sensor data was acquired using an Arduino Mega microcontroller development board (Arduino AG, Italy).

## Supporting information

S1 Fig Modifications done to the commercial cotton candy machine. **a** The wire screen that was used with the spinneret was replaced with an aluminium cylindrical ring of identical dimensions. Holes were drilled near the top edge of the cylinder to allow polymer melt to extrude through. **b** Installation of cardboard cover to hide the motor shaft in order to prevent spooling of extruded fibers by the motor shaft, thus allowing formation of a continuous sheet.
(TIF)

S2 Fig Fitration efficiency and pressure drop measurement testing setup. **a**, Filtration testing setup consisting of 1: Incense stick 2: Test filter assembly. 3: Lighthouse 3016 handheld particle counter **b**, schematic of est filter assembly where compressed sample is placed between two acrylic mesh screens, sealed on the sides with paraffin tape and held in place using the pipe screw setup.**c**, Pressure drop testing set up consisting of 1: Flow control valve 2: Airflow measurement sensor 3: Test filter assembly 4: Pressure sensor and micro-controller.
(TIF)

S3 Fig Morphological characterization of fibers. SEM characterization of of fibers produced from PP with 50 (**a, d**), 500 (**b, e**), and 1550 (**c, f**) MFI. Histograms (**g, h, i**) show distribution of fiber diameters obtained from SEM images in at least three separate locations.
(TIF)

S1 Video Modifed cotton candy machine in operation. Operation of repurposed cotton candy machine showing the use of recycled PP fibers to produce new layers of non-woven material.
(MP4)
