# Supplementary figures and images for: Low cost centrifugal melt spinning for distributed manufacturing of non-woven media
Source: PLoS One. 2022 Apr 19;17(4):e0264933. doi: 10.1371/journal.pone.0264933 (PMC9017944; doi:10.1371/journal.pone.0264933)

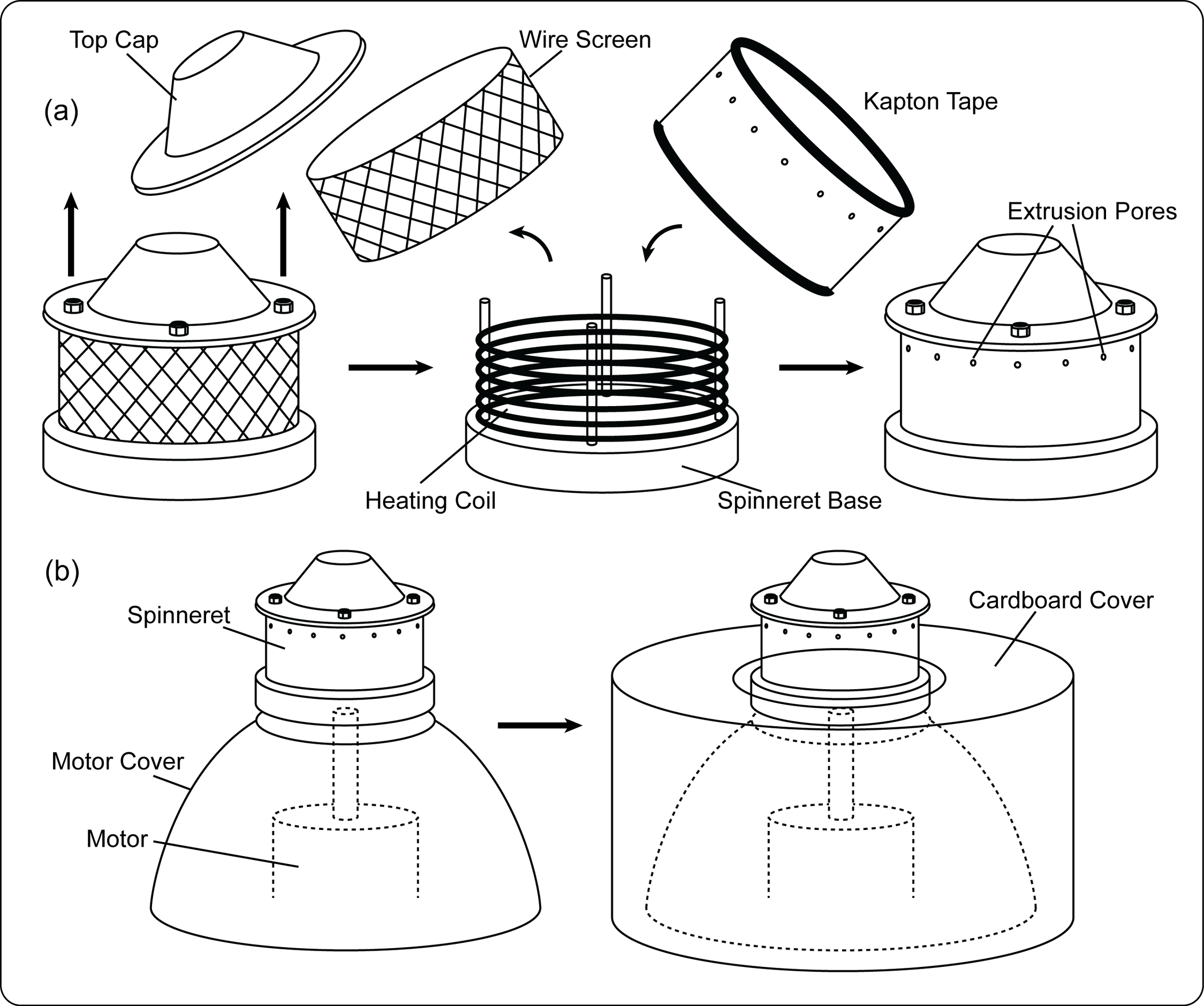

Supplement: S1 Fig — a The wire screen that was used with the spinneret was replaced with an aluminium cylindrical ring of identical dimensions. Holes were drilled near the top edge of the cylinder to allow polymer melt to extrude through. b Installation of cardboard cover to hide the motor shaft in order to prevent spooling of extruded fibers by the motor shaft, thus allowing formation of a continuous sheet. (TIF) [file pone.0264933.s001.tif]

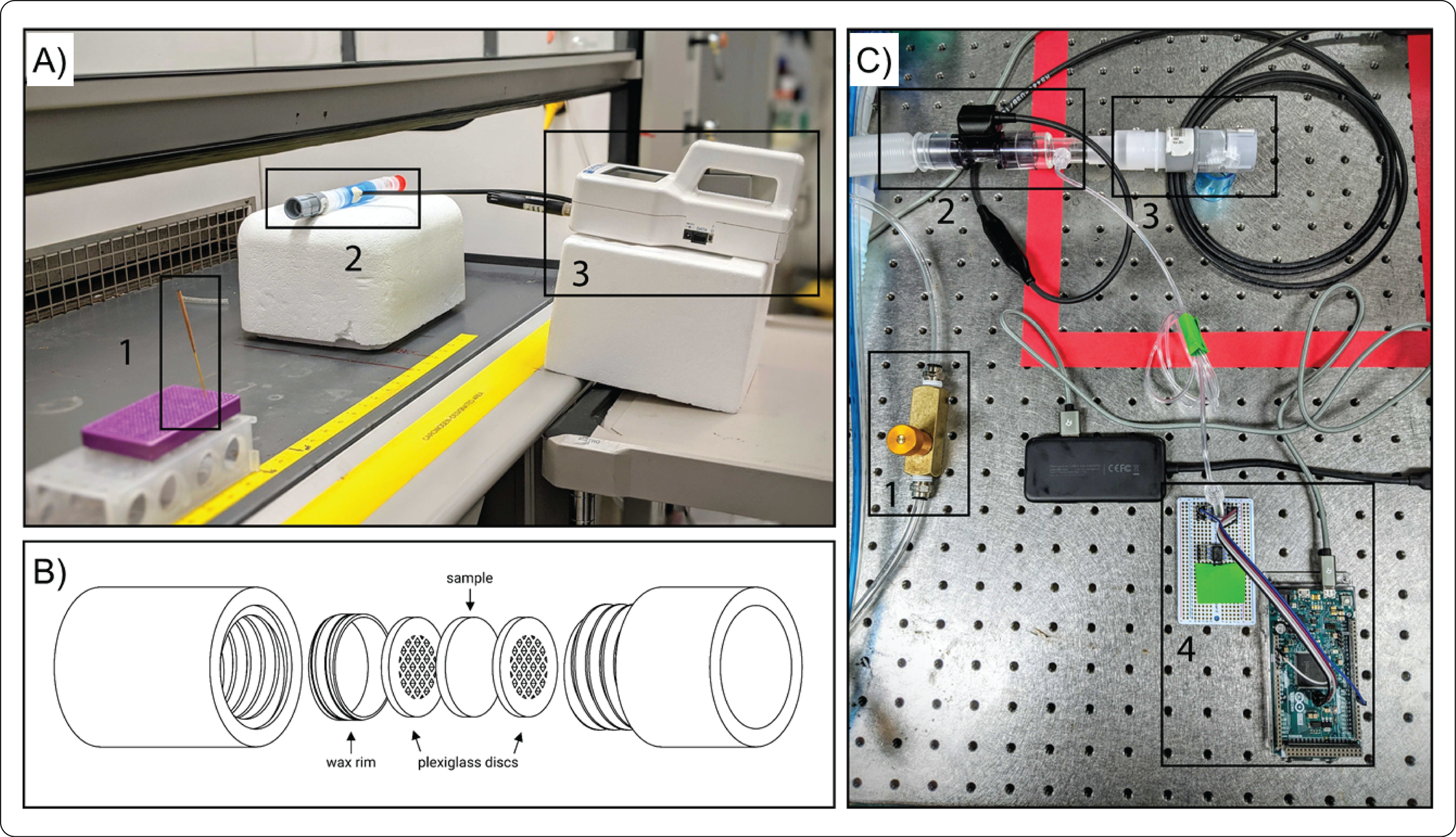

Supplement: S2 Fig — a, Filtration testing setup consisting of 1: Incense stick 2: Test filter assembly. 3: Lighthouse 3016 handheld particle counter b, schematic of est filter assembly where compressed sample is placed between two acrylic mesh screens, sealed on the sides with paraffin tape and held in place using the pipe screw setup.c, Pressure drop testing set up consisting of 1: Flow control valve 2: Airflow measurement sensor 3: Test filter assembly 4: Pressure sensor and micro-controller. (TIF) [file pone.0264933.s002.tif]

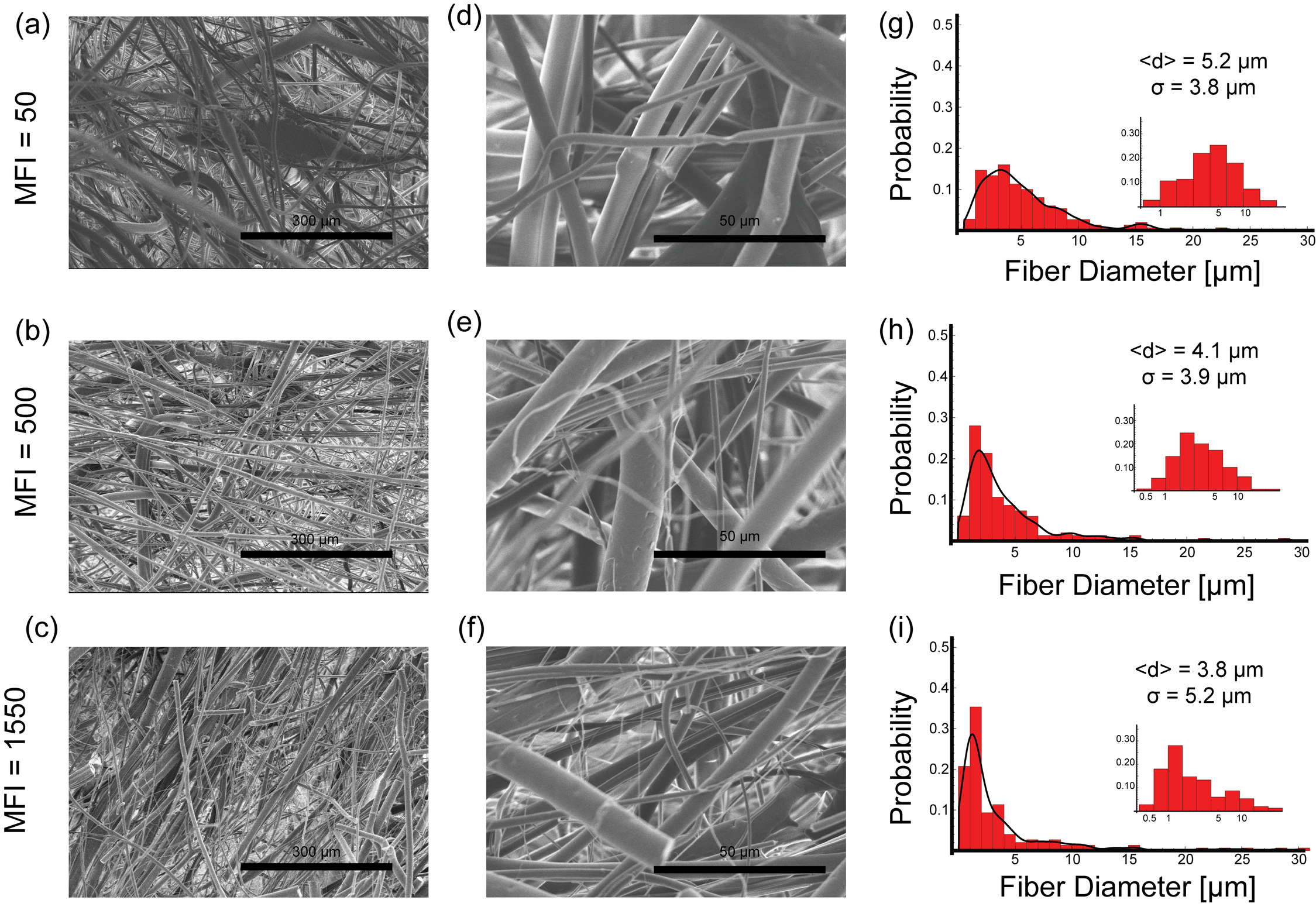

Supplement: S3 Fig — SEM characterization of of fibers produced from PP with 50 (a, d), 500 (b, e), and 1550 (c, f) MFI. Histograms (g, h, i) show distribution of fiber diameters obtained from SEM images in at least three separate locations. (TIF) [file pone.0264933.s003.tif]
